# Quo Vadis Particula Physica?

**DOI:** 10.3390/e26050366

**Published:** 2024-04-26

**Authors:** Xavier Calmet

**Affiliations:** Department of Physics and Astronomy, University of Sussex, Brighton BN1 9QH, UK; x.calmet@sussex.ac.uk

**Keywords:** standard model, quantum gravity

## Abstract

In this brief paper, I give a very personal account on the state of particle physics on the occasion of Paul Frampton’s 80th birthday.

It is a pleasure to contribute to this Special Issue on the occasion of Paul Frampton’s 80th birthday. I got to know Paul in 2004 when I moved from Caltech to UNC-Chapel Hill to take a postdoctoral position in his group. Paul very kindly came to knock at the door of my apartment on the day I arrived in Chapel Hill after an exhausting but amazing four and half days of driving from Pasadena to Chapel Hill. He took me to his place to have a chat and to show me the pictures of all the Nobel prize winners he had interacted with. I will never forget that Paul told me on that day that he had proposed so many extensions of the Standard Model of particle physics that he was bound to be awarded the Nobel Prize as one of his new particles would certainly be discovered at the CERN Large Hadron Collider (LHC). This was in 2004. The LHC discovered the Higgs boson in 2012. We are now in 2024 and sadly Paul is still waiting for the discovery of one of his particles and his prize. It is by now very unlikely that the LHC will be able to produce particles that are not part of the Standard Model, assuming they truly exist, at least not on-shell, see, e.g., [1].

To some, this lack of new physics beyond the Standard Model in the TeV region may not have been a surprise. My father, who was trained as a theoretical particle physicist in the 1960s, told me many times that his decision to move to computer science was strongly influenced by a discussion with Sheldon Glashow who was advocating in the 1970s the idea of a grand desert between the weak scale [2], i.e., the energy scale of the Standard Model, and the scale of unification at some $10^{16}$ GeV. My father, who was working on the heroic multi-loop calculation of the anomalous magnetic moment of the muon [3,4,5], became part of a small group of people who developed the field of computer algebra that was needed to perform these tedious calculations. He started a new research group in Grenoble in the mid-1980s, formally quitting physics and his original lab at Luminy in Marseille. He finally moved to the University of Karlsruhe, now KIT, in 1987, having accepted a professorship in computer algebra. There, he developed some contacts with the local particle physicists. In private, he would laugh at their effort to automatize Feynman diagram calculations in non-Abelian gauge theories including renormalization: he had done all these things in the 1970s and simply did not bother to publish them as they were trivial given his work on quantum electrodynamics. He had a very fulfilling career as a professor in computer science, never regretting having left physics.

What happened to physics since the 1970s? Well, first of all, people in the 1970s were too smart for the sake of my generation. The Higgs mechanism had been proposed by Peter Higgs [6,7] and embedded by Steven Weinberg in Sheldon Glashow’s electroweak model [8]. Harald Fritzsch and Murray Gell-Mann had proposed what turned out to be the correct theory for strong interactions (see, e.g., [9], for a review). Gerardus ’t Hooft and Martinus Veltman had shown [10] that these non-Abelian theories are renormalizable and thus mathematically consistent at all energy scales. This work enabled ’t Hooft to show that Yang–Mills theories can be asymptotically safe, long before the minus sign controversy and the following dilemma of attribution [11]. Sadly, for theorists trying to extend the Standard Model, experimentalists have found one particle predicted by the Standard Model after another, with the discovery of the Higgs boson being the last and final confirmation of the Standard Model.

Some will argue that neutrino masses are a clear sign of the breakdown of the electroweak Standard Model, but this is not something I find convincing. Neutrino masses are easily accounted for by the Standard Model if the Yukawa couplings between the left-handed neutrinos and right-handed neutrinos are not set to zero in full analogy to up-type quarks. There was never any real reason to set them to zero, besides the fact that their masses were compatible with zero given the experimental state of the art in the 1970s. I would see neutrino masses as a prediction of the Standard Model, because of the close analogy in the treatment of leptons and quarks in this model, rather than new physics. To a certain extent, this is a semantic question, but neutrino masses are not a theoretical challenge whichever point of view one takes.

As Glashow foresaw, it is thus conceivable that the Standard Model remains valid up to some very high energy scale, for example, the scale of grand unification, and threshold effects [12] could easily lead to the numerical unification of the gauge couplings of the Standard Model without the need for new physics between the weak scale and the grand unification scale (The issue of stability of the electroweak vacuum is an open one [13,14] as it depends on quantum gravity corrections.).

Despite Glashow’s insight, a few generations of physicists worked on so-called beyond-the-Standard-Model physics between the early 1970s and the late 2010s. This program was motivated by different reasons related to the question of the spontaneous breaking of the electroweak symmetry. In particular, the Higgs mechanism implied the existence of a fundamental scalar boson, something that had never been observed until 2012. This was a strong motivation to consider alternatives to the Higgs mechanism using the idea of dynamical symmetry breaking, where the scalar would effectively be a condensate of fermions. Technicolor and other composite Higgs models were very attractive from a theoretical point of view; sadly, it quickly became clear that the simplest and most elegant models were not compatible with data accumulated at colliders. Another logical possibility was that there could be a lot of fundamental scalars and not just the Higgs boson. This is the case of supersymmetric extensions of the Standard Model, where there are a minimum of two scalar fields for each fermion field depending on the amount of supersymmetry envisaged. Supersymmetry came with its own model-building issues; namely, these new scalar fields had to be made heavy to explain why they had not been discovered yet and supersymmetry had to be broken as we do not observe it as an exact symmetry of nature, at low energies at least.

So why did this program of looking for physics beyond the Standard Model fail so badly? On the one hand, one could argue that physicists of the age of Paul have been very unlucky; indeed, nature picked a model that was proposed when they were finishing their studies. There were good reasons to doubt the Standard Model. On the other hand, applying Gell-Mann’s criteria (one point for papers that are correct in the sense of being relevant to nature, minus one for papers that are not relevant to it) to evaluate particle physicists active in the last 50 years reveals that most of them are in the red and that they have been barking up the wrong tree.

Clearly one issue is that the main guiding principle to look for physics beyond the Standard Model was a red herring. Naturalness is the idea that the Higgs boson’s mass should be stable under radiative corrections. Proponents of this idea argue that the bare mass of a scalar field receives corrections at the quantum level from self-interactions and interactions with other particles of the model. These corrections are argued to grow quadratically with a dimensionful cutoff that is introduced to regularize loop corrections. They argue that if the cutoff is taken of the order of the reduced Planck mass (i.e., the energy scale where quantum gravitational effects are expected to become important), there needs to be some unnatural adjustment between the quantum corrections and the bare mass to keep the Higgs boson’s mass light. Most of the model building effort to go beyond the Standard Model has been motivated by this naturalness “problem”. Four broad classes of solutions have been envisaged: models without fundamental scalars, supersymmetric models, models with a low scale of quantum gravity and models advocating the anthropic principle.

In my view, naturalness is absolutely meaningless in the context of a renormalizable quantum field theory, as masses and coupling constants cannot be calculated from first principles. They are renormalized parameters which are used to absorb divergent quantities appearing in the perturbative evaluation of quantum amplitudes. As such, they need to be measured at some energy scale and can be scaled up or down using renormalization group equations, but as we cannot calculate these parameters from first principles, it is meaningless to talk about large or small values. Furthermore, whether divergences are quadratic or logarithmic plays no role from a physical point of view. One could also argue that the problem is not even well posed from a mathematical point of view, as the nature of the divergences depends on the regularization schemed used. For example, in dimensional regularization, quadratic divergencies do not appear in four dimensions.

It is remarkable that this problem was indeed first introduced by proponents of String Theory, where it is indeed possibly an issue as they claim to be able to calculate all fundamental constants which appear as the expectation values of some moduli fields in their framework. But it is certainly not an issue for particle physics. From a particle physics point of view, it should be clear to any researcher that the naturalness problem was not a valid guiding principle. The discovery of a light Higgs boson without new physics to stabilize its mass is the final nail in the coffin for naturalness after the discovery of a cosmological constant that is small and again without any new physics to stabilize it (similar arguments to those for the Higgs boson’s mass had been made for the cosmological constant). The particle physics community spent essentially 50 years trying to solve a problem which is not one.

It is fair to say that while experimental particle physics has been extremely successful for the last 50 years and found one particle of the Standard Model after the other, particle physics phenomenology has hit a wall and made very little progress partly because it has been guided by the wrong guiding principles.

Another issue that has affected theoretical particle physics overall is that because it has been increasingly disconnected from experimental physics, as it has been trying to solve a problem which is clearly not relevant to nature, it has become a beauty contest. An issue with beauty is that it obviously lies in the eye of the beholder and instead of applying Gell-Mann’s principle to evaluate scientists, less objective criteria have been applied, resulting in high-energy theory groups at top universities being taken over by people convinced that the single most important problem was the naturalness problem. Young people had to follow their lead and research to hope to be able to obtain a job in academia. The problem we are describing here is not unique to particle phenomenology, but it also applies to String Theory for the same reason: this program is mostly completely disconnected from experiment or to a certain extent from physics which is an empirical science. Overall, theoretical physics has become extremely speculative and the “cutest” speculations get rewarded with prestigious faculty positions and academic prizes.

As we have argued, physicists of Paul’s generation have been unlucky, but it is also clear that this generation decided to change the rules of the game when it became acceptable to invent new particles without being forced to do so by experiment or mathematical consistency of the theory.

How can my generation and younger theorists get out of this impasse? I can only offer a very personal opinion. We need to refocus research on what nature and mathematics are telling us. I see two clear problems, that while very difficult to solve, are certainly worth trying to address as they could guide us to an understanding of what lies beyond the Standard Model.

The first problem is obvious. It is dark matter, for which there is ample observational evidence. Unless all these uncorrelated observations are wrong, which would be very surprising, we know that Einstein’s theory of gravitation with visible matter (which can be described by the Standard Model) is not able to explain, e.g., the galaxy rotation curves or the Cosmic Microwave Background power spectrum. These phenomena are clearly fully disconnected and related to physics at different energy scales. However, they both point towards physics beyond the Standard Model, as no particle of the Standard Model can account for these observations. While a modification of gravitational physics is a logical possibility, it is unclear whether this would be sufficient to explain all observation such as, e.g., bullet clusters. The most logical explanation is clearly that there is some hidden sector of dark matter particles that is weakly interacting with itself and extremely weakly interacting with Standard Model particles, possibly only gravitationally (There is a caveat here as primordial black holes could account for a least a good fraction if not all of dark matter. I personally like this scenario very much as it does not require physics beyond the Standard Model.).

Here again, the guiding principle described above has led people to consider mostly a limited class of models called wimps, which stands for Weakly Interacting Massive Particles. Wimps are common in supersymmetric extensions of the Standard Model. Wimps are now mostly excluded by searches at LEP, Tevatron and the LHC. Again, there was no real theoretical reason to expect wimps to be relevant to nature, but it did not stop the field from making an industry out of these models. From a theoretical point of view, very little is known about the masses of dark matter particles and their interactions with regular matter. Without any serious theoretical prejudice or guidance, it seems unrealistic and unreasonable to build a new collider to exclude a small fraction of the allowed parameter range for dark matter models. There is a recent effort that appears very promising to me which consists of using existing quantum sensors to probe for ultra-light dark matter, see, e.g., [15]. These are cheap experiments, which are mostly already operating, e.g., atomic clocks, for other reasons. While they may not find dark matter, these experiments clearly have other important outcomes in, e.g., the field of quantum metrology, and quantum sensors have important practical implications which are likely to benefit humanity. There is thus a no lose game argument to be made for these experiments.

My suggestion to young theorists is to make an effort to talk to the atomic, molecular, and optical physics (AMO) community and to learn their slang. Progress in quantum technology is fast and there are plenty of opportunities to propose tests of the Standard Model using these new technologies based on quantum physics.

The other direction I would like to mention is that of quantum gravity. While we are still far away from having a theory of quantum gravity which is ultra-violet finite, modern quantum field theoretical techniques can be used to derive an effective action for quantum gravity that enables one to perform calculations for any physical process taking place at energies below the reduced Planck scale. This approach is called the unique effective action [16,17,18,19,20]. The only required assumption is that general relativity is the correct low-energy limit of the theory of quantum gravity. The effective action enables some model-independent predictions of quantum gravity (see, e.g., [21]). While these effects are, as expected, very small, they demonstrate that calculations in quantum gravity are feasible and do not require any speculation.

While these quantum gravitational effects are small and unlikely to be relevant to currently conceivable experiments, they can provide us with some important insights into quantum gravity. My hope is that this program could give us some hints about the correct fundamental theory of quantum gravity, for example, by providing us with consistency conditions.

I would like to emphasize that this approach has already produced some important results. Indeed, it has enabled us to show that black holes have a quantum hair, which is the key feature to explain how information escapes an evaporating black hole, thereby resolving the famous Hawking paradox [22,23,24,25,26]. It has also enabled us to calculate the leading-order quantum gravitational corrections to the entropy of a Schwarzschild black hole, which forced us to introduce the notion of quantum pressure for black holes [27].

I strongly believe that this is not the end of the story for this approach to quantum gravity. I believe that connecting our results to some ideas coming from String Theory such as the AdS/CFT correspondence or the Swampland program could help us discover interesting results connecting gauge theories and quantum gravity.

In terms of probing quantum gravity experimentally, I think that one should again turn towards quantum technologies. Establishing that gravity can entangle macroscopic objects would be highly interesting [28,29] and proof, if one is needed, that gravity is a quantum force.

My feeling is that we are making some important progress and that while the way people have performed particle physics for the last 50 years has to change, there are plenty of interesting opportunities for bright young theorists if they are willing to take some risks, ignore famous people and try to follow their physical intuition, mathematical consistency and nature.

Finally, let me argue that while the approach followed by Paul and this generation did not lead to new discoveries, it was still valuable in the sense that it pushed experimentalists to keep an open mind about the type of physics that could supersede the Standard Model. Paul with his creativity and productivity has played a crucial role in this endeavour. On this note, I would like to use this opportunity to congratulate Paul on his 80th birthday.

## Data Availability

This manuscript has no associated data. Data sharing not applicable to this article as no datasets were generated or analysed during the current study.
